# Injury to Southern Highbush Blueberries by Southern Red Mites and Management Using Various Miticides

**DOI:** 10.3390/insects11040233

**Published:** 2020-04-08

**Authors:** Lorena Lopez, Oscar E. Liburd

**Affiliations:** Entomology and Nematology Department, University of Florida/IFAS, 1881 Natural Area Drive, Gainesville, FL 32611, USA; oeliburd@ufl.edu

**Keywords:** tetranychids, mite injury, bronzing, blueberry plantings, Florida

## Abstract

Reports of severe infestations caused by southern red mites (SRM), *Oligonychus ilicis* McGregor (Acari: Tetranychidae), have increased in recent years in southern highbush blueberries (SHB). Currently, there is little known about the management of tetranychids in SHB, and only two miticides (fenazaquin and fenpyroximate) have recently been labeled for use in SHB. *Oligonychus ilicis* has caused up to 80%–100% losses in some blueberry plantings, and growers are looking for management tools for this new pest of blueberries. We report on injury to SHB from *O. ilicis* and the performance of seven miticides used to manage SRM populations, including spiromesifen, spiromesifen plus surfactant, vegetable oil concentrate, fenazaquin, “proprietary miticide” (referred to as Pro1), bifenazate, and fenpyroximate. Miticide efficacy was rated based on the number of SRM recorded on collected leaves and plant damage ratings using an arbitrary index (from 0 = no bronzing to 4 = 100% bronzing). Characteristic symptoms of leaf injury included purple or bronzed leaf color, leaf dryness and roughening. Fenpyroximate significantly reduced mite numbers three days after application. Additionally, plants treated with fenpyroximate or fenazaquin showed significantly less bronzing compared with the control plants. Overall, fenpyroximate and fenazaquin showed the best performance for the management of *O. ilicis* on SHB.

## 1. Introduction

Early ripening southern highbush blueberries (SHB) are hybrids of *Vaccinium corymbosum* L.×*V. darrowi* Camp (Ericaceae), and account for more than 90% of the production in Florida, which is estimated at more than 60 million USD [1]. Southern highbush blueberries are well adapted to mild winter climates and supply an early market at premium prices for blueberry growers [2,3].

Typically, major arthropod pests that attack SHB include thrips, *Frankliniella* spp. (Thysanoptera: Thripidae), spotted wing drosophila, *Drosophila suzukii* Matsumura (Diptera: Drosophilidae), and blueberry gall midge, *Dasineura oxycoccana* Johnson (Diptera: Cecidomyiidae) [4,5,6]. Prior to 2015, with the exception of the blueberry bud mite, *Acalitus vaccinii* Keifer (Acari: Eriophyidae), outbreaks of Acarine pests in SHB had never been reported. The first major tetranychid outbreak in Florida was in 2016 at a commercial operation where SHB was experimentally grown under protected structures [7]. Within the last three years, Florida and Georgia SHB growers have experienced severe losses and, in some cases, more than 200-ha have been left abandoned due to outbreaks of tetranychids. Damage estimates during 2019 range between 500,000 and 750,000 USD [8].

The southern red mite (SRM), *Oligonychus ilicis* McGregor (Acari: Tetranychidae), is also known as the red mite or the coffee red mite. It is an important spider mite pest, which feeds on more than 34 host plants from 15 families and causes economic damage to many ornamentals and fruit crops. These mites develop several overlapping generations each year during favorable conditions of relatively mild, dry and humid winters, when populations continue to grow without any diapause. They can complete one generation within two weeks under optimal conditions (25 ± 2 °C) on coffee and holly crops [9,10,11].

Southern red mites are now one of the main pest species attacking blueberry plants in the southeastern US. They feed on plant tissues by inserting their chelicerae into the host and removing cell contents, resulting in a net photosynthesis rate decline due to chloroplast destruction [12]. Southern red mites can reproduce on both the upper and lower surfaces of the leaf. Bronzed-colored leaves are the characteristic symptom associated with SRM injury in ornamental crops, and the intensity of the bronzing is proportional to the degree of internal leaf damage [12].

*Oligonychus ilicis* primary hosts include several ornamental plants such as camellias, azaleas, and hollies, whereas only a few fruit plants have been reported as hosts for this spider mite, including coffee, strawberry, and cranberry, the only reported host in the genus *Vaccinium* [9,10,11,12,13]. This paper reports highbush blueberry as a new host of *O. ilicis* from north-central Florida. Additionally, the evaluation of various newly registered miticides for the control of SRM in a commercial SHB planting, as well as information regarding leaf injury and plant damage, is included.

## 2. Materials and Methods 

### 2.1. Plant Culture 

A field trial was conducted between September 20th and October 22nd, 2019 at a commercial 303-ha blueberry farm located in Waldo, FL, USA (29°47′29.4648″N, 82°7′9.8832″W). Four 152-m long rows were randomly chosen from an SHB planting naturally infested with SRM. Bushes (cultivar #13123) were 1.5–2-m high and approximately two years old. Blueberry bushes were spaced 1-m apart, planted in single rows that were spaced 2-m apart, drip irrigated, and overhead irrigation was occasionally used. 

### 2.2. Leaf Injury Assessment

Damaged and undamaged leaves were initially photographed on the blueberry bush using an iPhone Xdigital camera. Subsequently, 15 injured (obviously bronzed) and 15 uninjured (green and healthy) leaves were collected from 10 randomly chosen untreated blueberry bushes. Leaf samples were placed in Ziploc bags and brought back to the Small Fruit and Vegetable IPM laboratory for processing. Pictures of injured and uninjured leaves were captured using an iPhone Xdigital camera and plant material was examined under a dissecting microscope. The number of SRM (adults and immatures) per leaf was recorded.

### 2.3. Performance of Miticides in Southern Highbush Blueberries

Eight treatments consisting of seven miticides and water (control) were evaluated as shown in Table 1. The experimental design was a randomized complete block with four replicates. Plots consisted of 12 bushes followed by eight untreated bushes used as a buffer zone between plots. One row of blueberry bushes was left untreated as a buffer zone between treated rows (~5-m apart). Two miticide applications were conducted (15-day interval) using a CO_2_ sprayer with Teejet hollow cone spray cores D3 disk DC 25 (Spraying systems Co., Keystone Heights, FL) and 1,380 L of water/ha for each application. No pesticides were applied within two weeks prior to the start of the trial. 

### 2.4. Collection of Samples and Plant Damage Assessment 

Mite population was assessed three days before miticide application (3-DBA), and three, seven, and 14 days after each application (DAA) for a total of seven sampling events (one pre-treatment, three, seven, and 14 days after the first application, and three, seven, and 14 days after the second application). Four bushes per plot were randomly chosen and sampled during the pre-treatment collection. To avoid repeated sampling after treatment, four randomly chosen bushes per plot were sampled, and tagged three and seven DAA. The remaining bushes were sampled 14-DAA. Fifteen leaves per blueberry bush were collected in 50-mL tubes and washed with 10-mL of 70% ethanol in the laboratory. Leaves were discarded and the ethanol containing the mites was checked repeatedly for adult and immature mites under a dissecting microscope. A representative sample of adult specimens (~30 mites) were slide-mounted for identification. 

Bronzing caused by mite feeding was rated on four randomly selected bushes 3-DBA and 14-DAA after the second application using an arbitrary plant damage index based on the percentage of bronzed foliage as follows: 0 = no bronzing; 1 = 1% ≥ 25% (low bronzing); 2 = 26% ≥ 50% (moderate bronzing); 3 = 51% ≥ 75% (high bronzing); and 4% = 76 ≥ 100% (severe bronzing). Blueberry bushes were rated by the same person each time, who examined the plant foliage thoroughly for bronzing symptoms.

### 2.5. Statistical Analysis 

The numbers of SRM (adults and immatures) were analyzed by fitting a generalized linear mixed model (GLMM). Data were fitted using a GLMM, as implemented in the PROC GLIMMIX procedure, following a negative binomial distribution to correct overdispersion when needed. This model considered the fixed effect factors of treatment, sampling event, and their interaction. In addition, the random effect of the block was considered. Plant damage data were analyzed by fitting a linear mixed model (LMM). Averaged indexes per plot were compared among treatments and sampling events (pre-treatment and 14-DAA) by using the PROC MIXED procedure. The LMM considered the fixed effect factors of treatment, sampling event, and their interaction, together with the random effect of the block. No transformation was used on any variable.

Comparisons of means among treatments at each sampling event for both GLMM and LMM were obtained by requesting the LSMEANS from each procedure, and the SLICE function for the effect of treatment when the GLMM was implemented. *p*-values less than 0.05 were considered significant. Lastly, Spearman’s rank correlation coefficients (r_s_) were used to assess the relationship between the number of mites and the plant damage index for pre-treatment and final measurements (14 days after the 2nd application). The PROC CORR procedure was implemented to obtain the coefficients, and Fisher transformation was used to calculate confidence intervals. All models were fitted using SAS 9.4 (SAS Institute, Cary, NC).

## 3. Results

### 3.1. Identification and Description of Leaf Injury

**Figure 1:**
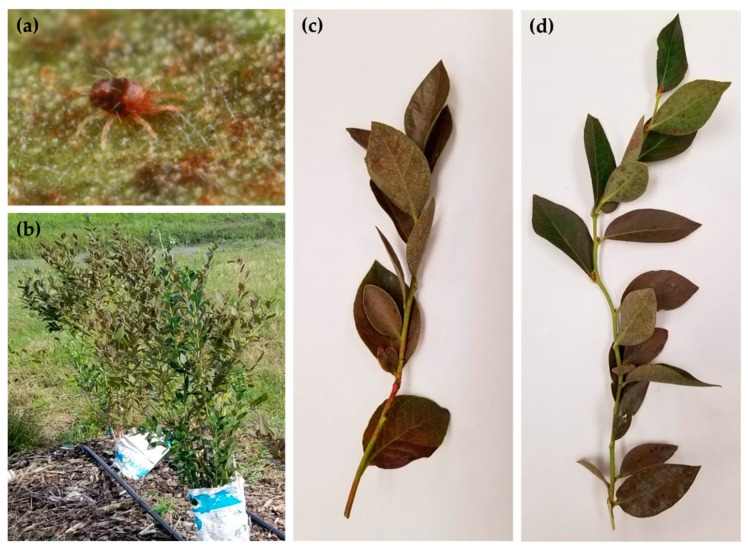
*Oligonychus ilicis* female on the lower side of a blueberry leaf (**a**), blueberry bushes with bronzing symptoms (**b**), blueberry leaves showing intense bronzing and accumulation of sheds on the lower side of the leaves (**c**,**d**). Photographs by Lyle Buss (**a**) and Lorena Lopez (**b**,**c**).

The mites were identified as southern red mites, *Oligonychus ilicis* McGregor (Acari: Tetranychidae), and confirmed by an acarologist taxonomist from the Florida Division of Plant Industry. *Oligonychus ilicis* were observed feeding and reproducing on the lower side of mature leaves, and moving upward as the population size increased (Figure 1a). The symptoms associated with injury caused by SRM were a characteristic purple or bronzed leaf color, leaf dryness and roughening, with whitish spots on the lower side of the leaves resulting from the accumulation of shed mite cuticles (Figure 1b–d). All of the injured leaves collected showed severe bronzing symptoms and the presence of *O. ilicis* was confirmed in 100% (6.21 ± 0.7 mites per leaf) of the injured leaves examined. Despite the absence of obvious bronzing symptoms, a few (1.02 ± 0.2 mites per leaf) SRM were found on the uninjured leaves examined.

### 3.2. Pre-treatment Counts 

Southern red mite (SRM) infestation levels observed at the beginning of the trial (3-DBA) averaged 4.8 (± 1.69) mites per leaf. Pre-treatment samples indicated that there were no significant differences among treatments.

### 3.3. Post-treatment Counts 

**Figure 2:**
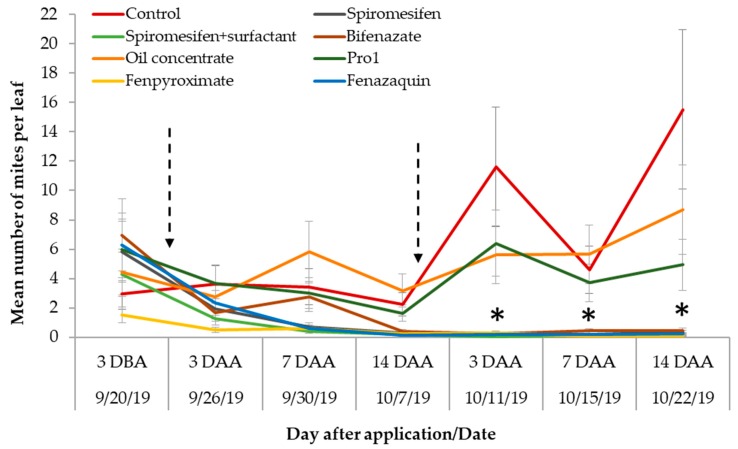
Mean (± SE) number of southern red mites per leaf recorded three days before miticide application (DBA), three, seven, and fourteen days after miticide application (DAA). Two miticide applications were conducted 15 days apart (on 9/23/19 and 10/8/19) represented here by the dotted arrows. Asterisks represent significant differences for mite numbers per leaf recorded on five treatments over time (Treatment*Sampling event interaction, F_42, 837_= 6.47; *p* < 0.0001) compared with control, oil concentrate and Pro1 treatments.

There was a significant treatment-by-sampling event interaction for the number of SRM per leaf (F_42, 837_= 6.47; *p* < 0.0001). Fifty percent of the miticides showed similar levels of mite infestation to those of the control plants three days after the 1st miticide application (3-DAA). *Oligonychus ilicis* numbers started to increase in the bushes that were treated with Pro1 and oil concentrate at 7-DAA and maintained an increasing tendency until the end of the trial (Figure 2). Most miticide treatments were successful at suppressing SRM after the 1st application on the blueberry bushes, including spiromesifen with and without surfactant, fenazaquin, and fenpyroximate. The mite numbers increased significantly over time in the blueberry bushes in the control treatment, as expected when pesticide treatment was absent (only water was applied) (Figure 2).

The blueberry bushes that were treated with spiromesifen alone (without surfactant) and fenazaquin showed a significant and continuous reduction in mite numbers, starting three days after the 1st treatment, and maintained infestation levels below two mites per leaf until the termination of the trial. The numbers of mites in bushes treated with bifenazate were reduced by approximately 70% (2 ± 0.6 mites per leaf) after the 1st application compared with the pre-treatment, and an even greater reduction was observed after the 2nd application (0.12 ± 0.048 mites per leaf). Bushes treated with fenpyroximate showed the lowest mite numbers throughout the trial, followed by spiromesifen mixed with surfactant (Figure 2).

### 3.4. Plant Damage Assessment 

**Figure 3:**
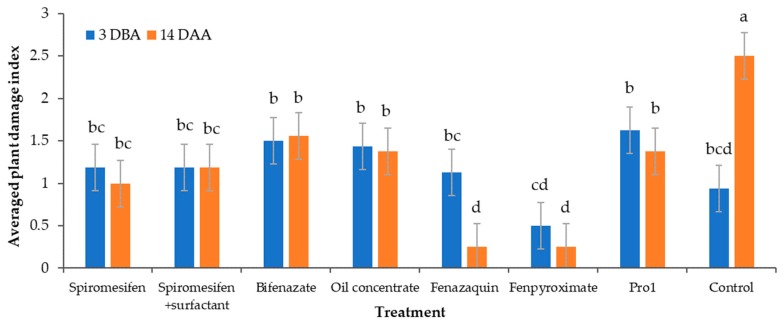
Bronzing of blueberry foliage caused by southern red mite feeding. Ratings taken pre-treatment 3 days before the 1st miticide application (3-DBA, on 9/20/19) and 14 days after the second and final miticide application (14-DAA, on 10/22/19) based on an arbitrary plant damage index (0 = no bronzing; 1 = 1% ≥ 25% (low bronzing); 2 = 26% ≥ 50% (moderate bronzing); 3 = 51% ≥ 75% (high bronzing); and 4 = 76% ≥ 100% (severe bronzing) bronzed foliage). Different letters across bars indicate significant differences (Treatment*Sampling event interaction, F_7, 237_= 3.63; *p* = 0.001).

There was a significant treatment-by-sampling event (pre-treatment and 14-DAA) interaction for the averaged plant damage index (F_7, 237_= 3.63; *p* = 0.001) (Figure 3). As expected, the percentage of bronzed foliage increased significantly in the control bushes, from ratings equivalent to the low bronzing recorded at the pre-treatment up to high bronzing symptoms at 14-DAA (0.93 ± 0.27 and 2.5 ± 0.27 averaged rating, respectively). 

The plant damage ratings recorded pre-treatment (3-DBA) were not significantly different among the treatments. Most blueberry bushes showed pre-treatment damage in approximately 25% of their foliage except for the bushes selected for fenpyroximate treatment, which had bronzed leaves in approximately 12% of their foliage (Figure 3). This is consistent with the low numbers of mites recorded during the pre-treatment counts in the bushes within the fenpyroximate plots (Figure 2). 

Most of the treatments showed little or no reduction in blueberry bronzing symptoms 14-DAA. The blueberry bushes treated with spiromesifen alone and spiromesifen mixed with surfactant remained in the low bronzing category, whereas bushes treated with bifenazate and oil concentrate were closer to reaching a moderate bronzing rating at 14-DAA. Alternatively, the blueberries treated with fenazaquin and fenpyroximate showed a 4.5- and a two=fold reduction over time in bronzing ratings, respectively (Figure 3). Fenazaquin and fenpyroximate treatments showed significant reductions in blueberry bronzing, consistent with the reductions in mite numbers recorded overtime and mentioned above (Figure 2).

**Figure 4:**
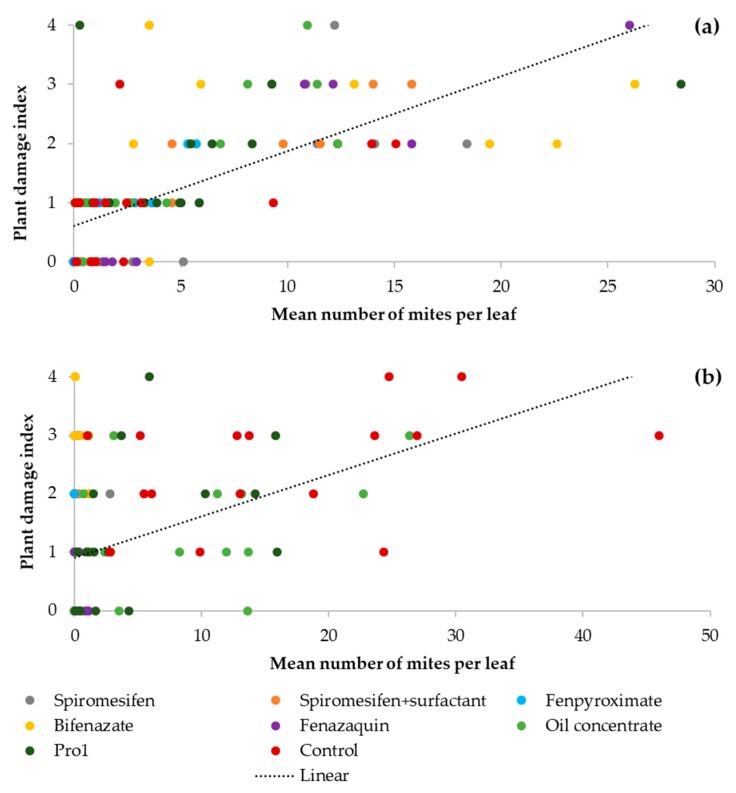
Relationship between the plant damage index and the number of mites per leaf for the pre-treatment (3-DBA) (**a**) and final measurements (**b**) at the end of the experiment 14 days after the 2nd and last miticide application (14-DAA). The plant damage index was used as follows: 0 = no bronzing; 1 = 1% ≥ 25% (low bronzing); 2 = 26% ≥ 50% (moderate bronzing); 3 = 51% ≥ 75% (high bronzing); and 4 = 76% ≥ 100% (severe bronzing) bronzed foliage.

There was a significant and strong correlation between the number of mites per leaf and the plant damage index for the pre-treatment counts (r_s_= 0.72; CI_95%_= 0.91, 0.63; *p* < 0.0001). Overall miticide treatments, an average of 4.58 (± 0.53) mites per leaf and low bronzing symptoms (index= 1.18 ± 0.10) were recorded in most of the blueberries sampled. The blueberry bushes hosting high numbers of *O. ilicis* mites showed increased bronzing symptoms at the beginning of the experiment, confirming that the bronzing observed in the blueberry foliage was a direct symptom caused by SRM feeding (Figure 4a). 

The correlation between the number of mites and the severity of plant damage continued to be significant at the end of the experiment, but a weak relationship was observed (r_s_= 0.45; CI_95%_= 0.30, 0.58; *p* < 0.0001). This is the result of the significant reductions in mite numbers (3.55 ± 0.60 mites per leaf) recorded in most miticide treatments at the end of the experiment, while the plant damage ratings overall treatments remained constant (index= 1.18 ± 0.19) within the four-week period of the trial (Figure 4b). 

## 4. Discussion

This study represents the first report of blueberries, specifically SHB (*V. corymbosum* L.×*V. darrowi*), as a host for SRM. Prior to 2015, *O. ilicis* was not detected during surveys carried out in Florida blueberries. However, high infestation levels were detected in the early fall of 2018 and 2019 in multiple areas of the commercial farm studied in Florida, as well as in one commercial farm in Georgia where approximately 40-ha were severely damaged by SRM populations [7]. 

Leaf injury caused by *O. ilicis* varies based on the host plant. In strawberry, camellia, and cherry laurel, SRM feeds on both the lower and upper parts of the leaf causing stippling, and gray or bronzed color [12,14]. The southern red mite feeds and reproduces in the upper side of the leaf on coffee plants, causing yellowing, bronzing, and leaf roughening as the populations increase [12,15]. Similar to coffee, no stippling symptoms were observed in injured blueberry leaves during this study; however, SRM were observed infesting only the lower side of the blueberry leaves. The main symptoms of SRM damage in SHB were rapid and intense bronzing, leaf roughening, and accumulation of shed cuticles. 

There was a strong relationship between bronzing symptoms and SRM presence before treatment with miticides, confirming that the bronzing symptoms were caused by infestation with SRM and not by abiotic or other biotic factors. The presence of a few mites on uninjured leaves may be the result of initial infestation processes. Additionally, plant recovery was observed in blueberries treated with miticides such as fenazaquin and fenpyroximate, which showed a rapid and substantial suppression of mite populations (4.5- and two-fold reductions, respectively). These bushes showed a rapid growth of new flushes in the three weeks following the 1st miticide application, demonstrating the recovery capacity of SHBs after infestations with SRM. Plant recovery was not observed in any of the other treatments, where most damage ratings remained constant. Thus, the weak correlation observed at the end of the experiment may be explained by substantial reductions in mite numbers in only a couple of treatments, while most plant damage ratings remained unchanged.

*Oligonychus ilicis* is one of the major pests in coffee plantations in South America, and the main control method adopted by coffee producers is the use of miticides. Initial infestations of 15–120 females of *O. ilicis* per leaf can result in a significant decrease in photosynthesis, causing substantial damage to coffee crops. Therefore, miticides are sprayed early and frequently throughout the season for severe infestations [11,16]. Blueberries appear to be more sensitive to SRM injury when compared with coffee, showing bronzing symptoms (~25% of bronzed foliage) with as low as six mites per leaf. By the time that populations increased to 15 mites per leaf, the blueberry bushes showed high bronzing symptoms in up to 75% of their foliage. These numbers fall far below the action thresholds established for other spider mites such as the two-spotted spider mite (*Tetranychus urticae* Koch, Acari: Tetranychidae) in strawberries, where ≤ 80 *T. urticae* per trifoliate leaf indicates the time for miticide applications [17]. Low numbers of mites associated with highly bronzed bushes may also be the result of mite populations moving into new healthy plants after heavily damaging previous bushes. However, there is no available information on the damage thresholds or moving patterns of SRM in blueberries to confirm these hypotheses.

Until 2018, no miticides were registered for use in highbush blueberries. Fenazaquin was registered for use in highbush blueberries in 2019. Thus, it was used as the standard miticide during this study. The numbers of SRM declined slowly after the 1st treatment with fenazaquin, and were significantly suppressed after the 2nd application. A similar pattern of decline was observed in bushes treated with bifenazate. These miticides provide 3–5 weeks of residual control [18,19], and the slow decline in mite numbers may be related to their residual effect [20]. Residue trials for bifenazate in blueberries are currently being pursued by Interregional Research Project No. 4 (IR-4), and this chemistry holds potential for future registration in blueberries. 

Fenpyroximate is labeled for low growing blueberry bushes and just received registration in 2020 for use in highbush blueberries. Yet, the southern red mite is not listed as a target pest on the fenpyroximate and the fenazaquin label. Similar to fenazaquin, fenpyroximate and spiromesifen mixed with surfactant successfully suppressed SRM populations after the 1st treatment, showing good potential for mite management in blueberries. The remaining products tested are not yet registered for use in blueberries, but are under the evaluation process for future registration. 

There is little information regarding SRM damage in blueberries and no management tools are available to address this risk. Only a few studies have investigated the efficacy of plant protection products on SRM, and most of this research is outside the US. [21,22,23,24]. Similarly, only a few authors have evaluated the potential of biological control agents for management of SRM. The predatory mite *Euseius alatus* DeLeon (Acari: Phytoseiidae) has been reported as effective in suppressing SRM populations in coffee crops [16], and phytoseiid mites such as *Typhlodromus pyri* Scheuten and *E. finlandicus* Oudemans have been recorded in blueberry plantings in Europe [25]. However, data on the occurrence of these predatory mites in blueberries are lacking.

## 5. Conclusions

This research documented, for the first time, SRM injury on blueberries and provided a list of miticidal tactics that can be employed to suppress the SRM population. The reasons behind the SRM outbreaks in Florida and Georgia blueberries are not yet clear. We hypothesize that an increased reliance on broad-spectrum insecticides for the control of spotted-wing drosophila (*D. suzukii*) during blueberry harvest, and post-harvest sprays with pyrethroids for control of the blueberry leaf beetle (*Colaspis pseudofavosa* Riley, Coleoptera: Chrysomelidae) [4], have had a detrimental effect on natural enemy species that can regulate mite pests in the blueberry crops. In addition, there is evidence of pyrethroid-induced outbreaks of SRM, where the exposure of mites to low concentrations of pyrethroids has resulted in increased populations [22]. This combination of factors may have led to the outbreak of a new mite pest in southern highbush blueberries. However, additional studies are needed to test these hypotheses. 

Finally, establishing action thresholds is vital to design management programs for SRM, in addition to evaluating the performance of miticides with some degree of selectivity to avoid non-target effects. Likewise, reliance on broad-spectrum insecticides for other major pests in blueberries would need to be reduced in order to consider biological control approaches in the future.

## Figures and Tables

**Table 1:** List of miticide products tested and the label recommended rate used for control of *Oligonychus ilicis.*

Treatment (Active Ingredient (AI))	Miticide Product (Brand Name)	Product Rate: AI/ha	Manufactory
Spiromesifen	ALPB2017	1.25-L/ha	Bayer, St. Louis, MO
Spiromesifen + vegetable oil as surfactant agent	ALPB2017 + Dyne-Amic^®^	1.25-L + 2.5% v/v	Helena Agri-Enterprises, LLC, Collierville, TN
Bifenazate	Acramite^®^	1.12-kg	Arysta LifeScience, LLC, Cary, NC
Vegetable oil concentrate	Agridex^®^	4.75-L	Helena Agri-Enterprises, LLC, Collierville, TN
Fenazaquin	Magister^®^	2.65-L	Gowman Co., Yuma, AZ
Fenpyroximate	Portal^®^	2.38-L	Nichino America, Inc., Wilmington, DE
Proprietary miticide	Referred to as Pro1	1-L	NA
Control (water)	NA	NA	NA

NA: not applicable.
